# A novel screening tool (Karanth’s test) for vitamin B12 deficiency: a pilot study

**DOI:** 10.1186/s13104-015-1744-1

**Published:** 2015-12-12

**Authors:** Veena K. L. Karanth, Laxminarayan Karanth, Tulasi K. Karanth, Sowmyashree K. Karanth, Ragini Bekur

**Affiliations:** Department of General Surgery, Kasturba Medical College, Manipal, Udupi, India; Department of Obstetrics and Gynaecology, Melaka Manipal Medical College, Melaka, Malaysia; Kasturba Medical College, Manipal, Udupi, India; Department of Internal Medicine, Kasturba Medical College, Manipal, Udupi, India

**Keywords:** Vitmain B12, Von Luschan skin tone chart, Screening

## Abstract

**Background:**

No practical tests are currently available for screening vitamin B12 deficiency because the available techniques are invasive, expensive, and require a particular level of infrastructure and service that is not available in all places such as rural areas. Thus, we have examined the efficacy of a novel method (Karanth’s test) for identifying people with vitamin B12 deficiency as part of a pilot study.

**Methods:**

An observer-blind study was conducted on 83 consenting patients from a tertiary teaching hospital whose blood was drawn for estimation of serum vitamin B12 over a 2-month period. All of these patients completed the study. In the Karanth’s test, the skin color tone is measured at the interphalangeal joint and the phalanx using the Von Luschan skin tone chart. The test result is obtained from differences in the values obtained. This test was performed on the day blood was drawn to measure the serum vitamin B12 levels in the study patients and on every day until discharge for patients tested to be deficient.

**Results:**

Of the 83 patient subjects, 20 showed deficient vitamin B12 levels in the blood test. The Karanth’s test readings were significantly different for patients with normal and deficient levels of vitamin B12 (95 % CI, 0.838–2.153). ROC curve analysis suggested that a difference greater than 1.5 should be considered positive. The sensitivity and specificity of the test were determined to be 80 and 84.1 %, respectively. Patients were grouped further according to the Fitzpatrick scale. There were no type I, II or III patients and insufficient IV cases to determine sensitivity and specificity. Sensitivity and specificity were determined to be 57.1 and 94.6 % in type V and 92 and 63.6 % in type VI, respectively. We found that 87 % of our patients who tested positive had normal values on discharge.

**Conclusion:**

The Karanth’s test is a useful screen for a vitamin B12 deficiency and warrants further evaluation in a larger study population.

**Electronic supplementary material:**

The online version of this article (doi:10.1186/s13104-015-1744-1) contains supplementary material, which is available to authorized users.

## Background

Vitamin B12 is a water-soluble vitamin produced mainly by the action of microorganisms in the intestine. Animals obtain vitamin B12 either through the action of their own internal natural bacterial flora or by consuming other animals. Humans can do so be consuming pigs, oysters, shrimp, and chicken. Vegetarians get their share of vitamin B12 only from milk and its derivatives [1].

The major manifestations of vitamin B12 deficiency are hematological and neurological. Vitamin B12 is required for DNA maturation and is thus first manifested in rapidly dividing cells, namely the blood cells. This impact on blood cells can be best observed by performing a peripheral smear. The first manifestation of a vitamin B12 deficiency is a hyper-segmented neutrophil nucleus. Later, megaloblastic anemia develops, in which the cells are enlarged and the levels of hemoglobin are reduced. Clinically, the affected patients will present with symptoms of anemia and will predominantly develop weakness, fatigability, palpitations, and tachypnea. The common neurological manifestations of vitamin B12 deficiency include tingling and numbness in the extremities, weakness, motor disturbance, vision loss, and behavioral changes such as dementia, subacute degeneration of spinal cord, hallucination, psychosis [2–4] and depression. Importantly, patients already suffering from neurological diseases such as Alzheimer’s or Parkinson’s disease [5–7] will develop enhanced symptoms of these disorders if they become vitamin B12 deficient and only a minority will recover after treatment [8].

Loss of appetite, constipation, diarrhea, flatulence, and glossitis are some of the gastrointestinal manifestations that can occur with a vitamin B12 deficiency. Cutaneous manifestations also occur early in vitamin B12 deficiency and present as skin hyperpigmentation, vitiligo, angular stomatitis, and hair changes [9]. Hyperpigmentation in such cases is characteristically more evident at the knuckles and interphalangeal joints than at the phalanges [9, 10] for unknown reasons.

**Fig. 1:**
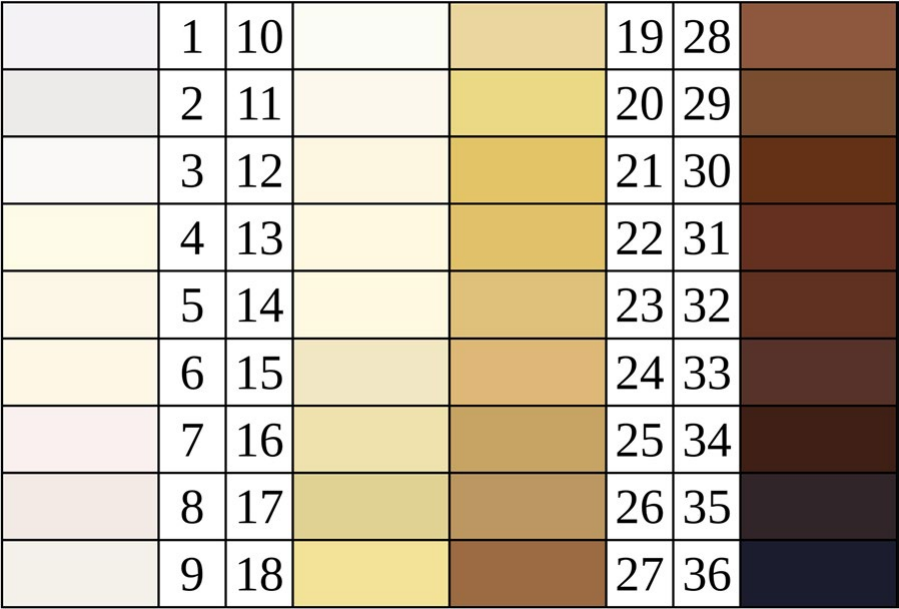
Von Luschan skin tone chart

No practical screening tests are currently available that will identify a vitamin B12 deficiency. The diagnosis of a vitamin B12 deficiency at present requires the relevant clinical expertise and expensive laboratory investigations [11]. In our current study, we tested a novel method as a more practical screen for this deficiency. In this test, the degree of hyperpigmentation over the interphalangeal joint compared with the phalanges is measured to determine the vitamin B12 status. The Von Luschan skin tone chart (VLSTC; Fig. 1) has been used previously to measure hyperpigmentation [12] and comprises 36 shades that cover all known skin colors around the world. These color shades are arranged in an ascending order of darkness with number 1 representing the fairest skin possible and 36 the darkest skin possible. A printed version of the VLSTC was used in our analyses to determine the efficacy of our new screening method for vitamin B12 deficiency in a small study population. We have named this method the Karanth’s test, and a patent application is pending.

## Methods

### Study population

The present study was conducted on patients admitted to the general medicine ward of a tertiary teaching hospital Kasturba Medical College and Hospital, Manipal, India, in the months of July and August 2013. Patients admitted to the general ward whose blood was taken for serum vitamin B12 estimation were included in the study and a written informed consent was taken. Those patients who failed to give consent or were critically ill (e.g., if they were in the intensive care unit) were excluded. Approval for this study was obtained from Institutional Ethical Committee of Kasturba Medical College and Hospital, Manipal University.

### Karanth’s test

The Karanth’s test was performed on a series of patients who were receiving a blood test for vitamin B12 prior to obtaining these laboratory results. The laboratory results therefore had no influence on finger color evaluations. Briefly, the person to be screened is taken to a natural light source and is made comfortable. The hand of the patient, preferably the left, is held out. A printed version of the VLSTC is compared with the skin over the proximal interphalangeal joint of the index finger and the corresponding reading is noted. Next, the VLSTC is compared with the skin over the middle phalanx of the index finger and the corresponding reading is noted. The second reading is subtracted from the first reading. The difference between the two readings is taken as the result. As this study was performed to evaluate the efficacy of the Karanth’s test, cut-off values were not determined during the data collection. This was performed on the day blood was taken for serum vitamin B12 measurement and on subsequent days for patients who tested positive.

### Serum vitamin B12 measurements

The vitamin B12 levels were determined in blood samples from each study subject via a chemiluminescence technique with a laboratory-defined cut-off value (based on unpublished study on population attending our hospital) of 180 pg/dl. Thus, patients with serum vitamin B12 levels lower than 180 pg/dl were considered vitamin B12 deficient. The laboratory personnel were blind to the results of the Karanth’s test.

### Grouping

Two individuals were involved in data collection. One noted the laboratory values and classified patients as deficient or non-deficient. The other performed the Karanth’s test and noted the readings. Neither researcher was aware of the other results. The patients were grouped into the ‘low B12’ group if their serum vitamin B12 showed a deficiency. The remainder were grouped as ‘normal B12’. The patients were further grouped into one of six Fitzpatrick types.

### Statistical analysis

Statistical significance was determined using the Student’s *t* test (*P* < 0.05). An ROC curve was plotted to obtain the cut-off value for Karanth’s test. Sensitivity and specificity were calculated based on this cut-off value. Sensitivity and specificity were also calculated for the six Fitzpatrick skin types to look at how Karanth’s test performs in different skin tones. Percentage of normalization on discharge was calculated for those who were deficient and tested positive in the Karanth’s test (Additional file 1).

## Results

The study was conducted from 1 July to 31 August 2013. Of the 83 participants, 20 were in the low B12 group (7 women and 13 men) and 63 were in the normal B12 group (29 women and 34 men). The mean age was 49 years (range 20–77); two patients were younger than 21 years, 46 were aged between 22 and 50 years, and 35 were older than 51 years. The time interval between the collection of the blood samples for vitamin B12 estimation and the performance of Karanth’s test was less than 24 h. No treatment was administered between these tests. There were no adverse events resulting from the Karanth’s test as it is noninvasive.

**Fig. 2:**
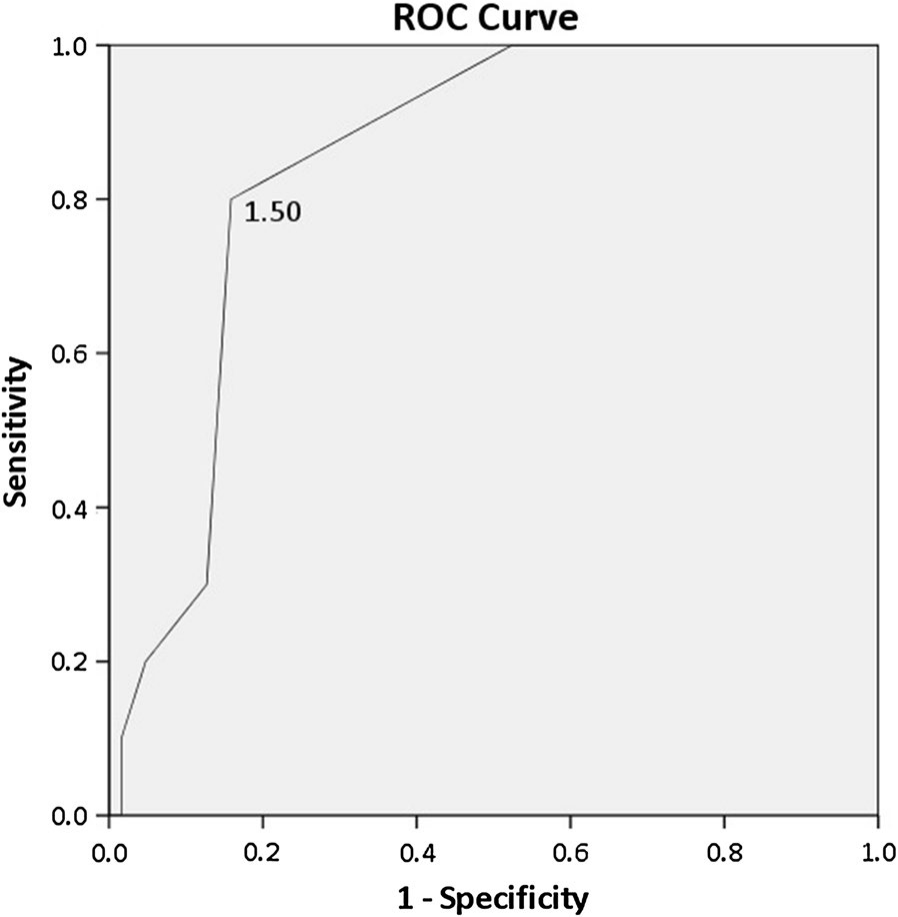
ROC curve. The optimal sensitivity and specificity were obtained when a difference of greater than 1.5 between the scores for the two skin regions was taken as positive

The Karanth’s test scores were significantly higher in the low B12 group than in the normal B12 group (95 % CI, 0.838–2.153; *P* < 0.001). ROC curve analysis (Fig. 2) showed that a score difference between the two skin regions of larger than 1.5 (i.e., two or more, as readings obtained after the Karanth’s test cannot be in decimals), produced the best sensitivity and specificity for the test. Therefore, the cut-off for normal individuals was taken to be 1. Using a reading of two or more as a positive test result (vitamin B12 deficient) yielded the following results: true positives (TPs)—a vitamin B12 deficiency indicated by both Karanth’s test and serum vitamin B12 levels (n = 16); true negatives (TNs)—no vitamin B12 deficiency according to both Karanth’s test and serum vitamin B12 levels (n = 53); false positives (FPs)—a vitamin B12 deficiency indicated by the Karanth’s test but not by the serum vitamin B12 levels (n = 10); and false negatives (FNs)—no vitamin B12 deficiency according to the Karanth’s test but low serum vitamin B12 levels indicated in the blood test (n = 4). Sensitivity [TP/(TP + FN)] and specificity [FP/(TN + FP)] values of 80.0 and 84.1 %, respectively, were obtained. Karanth’s test showed predominantly positive results for vitamin B12 levels below 150 pg/dl, mixed results up to 300 pg/dl and predominantly positive results for levels above that.

There were no patients grouped in Fitzpatrick type I (very light), type II (light), type III (light intermediate). The number of patients grouped as type IV (n = 4) was insufficient to calculate sensitivity and specificity. The sensitivity and specificity was 57.1 and 94 % for type V (low B12 = 7, normal B12 = 37) and 92 and 63.6 % for type VI (low B12 = 13, normal B12 = 22) cases, respectively.

When we excluded four cases in our series who showed a normal difference even though they were vitamin B12 deficient, 14 of our test patients had a difference of 0 and 1 at the time of their discharge. Therefore it was calculated that 87 % of our study subjects had reached normalcy at discharge.

## Discussion

Screening for vitamin B12 deficiency is very important as it is a recognized health issue and has been widely studied. If detected early, the manifestations of a vitamin B12 deficiency are reversible, but certain late-stage symptoms are irreversible. The prevalence of this disease is high in some countries such as India (80 % of Indian preschoolers and 70 % of Indian adults have a marginal or deficient status [13]) mainly due to an increasing elderly population, a low socio-economic status of a large part of the population which contributes to malnourishment, the popularity of vegetarianism for cultural reasons, and the decreased consumption of milk and milk products resulting from lifestyle changes. In our present study, the average age of the participants was 49 years and the prevalence of a vitamin B12 deficiency 19.27 % among these subjects.

Vitamin B12 deficiency has been classified into various stages, from asymptomatic to late [14]. This staging helps to determine the prognosis of the affected patient and the identifying test that should be used. The use of Karanth’s test enables at-risk individuals to be quickly identified so that they can undergo detailed clinical and laboratory investigations to confirm their vitamin B12 levels. There are effective treatments for a vitamin B12 deficiency such as daily oral tablets and periodic injections, both of which are usually accepted by the affected patients. Earlier interventions can reverse the effects of the deficiency, especially neurologic changes, that would eventually become irreversible if left untreated.

The early asymptomatic stages of vitamin B12 deficiency are readily recognizable. These include cutaneous manifestations such as hyperpigmentation, especially over the interphalangeal joints, which allow early identification of the disease. The mechanism by which a vitamin B12 deficiency causes hyperpigmentation is not yet known, but it is thought to involve the stimulation of melanocyte growth. This feature is the basis of the Karanth’s test and will likely prove to be particularly useful in the screening of large populations. As we here demonstrate, this test is effective and it may help clinicians to reduce the incidence of dangerous consequences of overt vitamin B12 deficiency, such as anemia and subacute degeneration of the spinal cord. It may also be possible using this practical test to reduce the costs associated with untreated vitamin B12 deficiencies to the health systems of the most affected countries by identifying possible cases far more quickly. The more costly serum vitamin B12 blood tests, and other invasive and time-consuming tests, can then be performed on a more targeted group of individuals with a suspected vitamin B12 deficiency. The VLSTC is inexpensive and skin color tone matching is easy to perform, making it a good screening approach. The Karanth’s test showed a sensitivity and specificity of 80.0 and 84.1 %, respectively, in our current study. As our participant population was small however, these values will need to be recalculated using a larger population in the future.

There were no adverse events associated with the Karanth’s test as it is noninvasive. Included among the many advantages of this test is that it is inexpensive to perform, with one easily available chart being sufficient for many people. In addition, minimal expertise was found to be required to perform the test and interpret the results. One possible disadvantage of this test to note is that is has a degree of subjectivity: two examiners may produce different readings for the same person at the same time. This problem can be largely overcome however by the fact that the results depend on the differences between two readings and not on individual readings.

As the Karanth’s test produced predominantly positive results in cases with vitamin B12 measuring less than 150 pg/dl, we contend that it will be able to identify most of the deficient cases even in set-ups using different cut-off values. As population was not equally distributed in the six Fitzpatrick types [15] in our study series, this test needs to be verified in a larger population, with cases distributed equally among the six Fitzpatrick types, to determine how this skin test will perform in populations with different skin tones.

## Conclusion

The Karanth’s test is a very useful and practical screen for vitamin B12 deficiency in a large group of patients without the need for expensive and complex equipment. This test shows great potential and warrants further evaluations in larger cohorts.

## Additional file

10.1186/s13104-015-1744-1 STARD Checklist.
